# Lateral approach is advantageous in total knee arthroplasty for valgus deformed knee

**DOI:** 10.1007/s00590-012-1137-2

**Published:** 2012-11-21

**Authors:** Hitoshi Sekiya, Kenzo Takatoku, Hisahi Takada, Naoya Sugimoto, Yuichi Hoshino

**Affiliations:** Orthopaedic Surgery, Jichi Medical University, 3311-1 Yakushiji, Tochigi, Shimotuke 3290498 Japan

**Keywords:** Valgus knee, Total knee arthroplasty, Lateral approach, Postoperative flexion

## Abstract

**Introduction:**

For the total knee arthroplasty in valgus deformed knee, superiority of the medial or lateral approach is still controversial. We compared the short-term result of two approach groups.

**Materials and methods:**

Forty-seven knees in rheumatoid arthritis with valgus deformity from 6° to 24° were randomly divided into two group; medial approach (24 knees) and lateral approach (24 knees). We used Scorpio NRG PS for all knees. Median postoperative periods were 43 months in both groups. We compared the surgical time, and alignment on standing radiograph, range of motion (ROM) pre/postoperatively, and degrees of soft-tissue release procedure, and lateral laxity measured by stress radiograph immediately after operation and at final follow-up.

**Result:**

Pre/postoperative alignment, surgical time, lateral laxity, and preoperative ROM had no significant in two groups; however, postoperative flexion was superior in lateral approach group 123.8°, 109° in medial approach group. All cases required iliotibial band (ITB) release at Gerdy’s tubercle, 83 % ITB at joint level, 21 % lateral collateral ligament (LCL), 17 % popliteus tendon (PT) in medial approach group, and 88 % ITB at Gerdy’s tubercle, 46 % ITB at joint level, 13 % LCL, 4 % PT in lateral approach group.

**Discussion:**

In the valgus knee, lateral structures are tight. Lateral approach can directly adjust the tight structure, and also less vascular compromise to the patella than medial approach with lateral patellar release. Less invasiveness to the quadriceps muscle in lateral approach could result into better range of motion after the surgery.

## Introduction

Nowadays, total knee arthroplasty (TKA) is a well-established procedure and has proven to be durable and effective for the treatment of advanced arthritis of the knee joints; however, the long-term results in valgus deformed knee were relatively inferior to those of varus deformed knee. One of the reasons of poor prognosis might be the difficulty to acquire good soft-tissue balance during the surgery. In treating valgus deformity of the knee by TKA, sequential releases of soft tissue on the lateral side of the knee are usually necessary, including the iliotibial band (ITB), lateral patellar retinaculum, lateral collateral ligament (LCL), and at times the popliteus tendon (POP), lateral gastrocnemius tendon, and biceps femoris tendon [1]. Some authors [2–4] have stated that releases of these structures are best addressed by direct access using a lateral approach.

Good results had been reported using this approach [3]. Some authors have reported poor result for TKA in valgus deformed knee using a conventional medial approach [3, 5, 6]. In addition, vascular compromise after TKA by a medial approach with lateral retinacular release had been reported [2, 7, 8]. But for most orthopedic surgeon, the lateral approach was not familiar technique to perform. Osteotomy of the tibial tubercle was usually necessary, and many complications were also reported with the procedure. Ranawat et al. [9] reported good results using a medial approach with lateral retinacular release. There were several reports which compared the clinical results between medial and lateral approach with the osteotomy of tibial tubercle [10, 11]; however, superiority of the medial or lateral approach still remains controversial. To solve this problem, we prospectively compared short-term results of two approach groups.

## Materials and methods

Inclusion criteria of the study as follows: primary unilateral TKA, female, rheumatoid arthritis, valgus deformity from 6° to 24°, preoperative flexion contracture <31°, and preoperative flexion angle >89°.

Forty-eight cases were included in this study. Each case was randomly divided into two groups: group of medial approach (n = 24); and group of lateral approach (n = 24). Mean age of the all cases was 65 years (range, 50–77 years) at the time of surgery. Details of the groups are shown in Table 1. No difference was found in age, height, weight, preoperative mechanical axis, preoperative range of motion (ROM), and preoperative knee society score (KSS) between two groups. A medial parapatellar approach was used for the medial approach, and a lateral parapatellar incision without osteotomy of the tibial tubercle was applied for the lateral approach. There were several surgical tips for successful lateral approach without osteotomy. One was the incision to the quadriceps tendon from superficial lateral to deep medial, allowing for natural expansion of the laminated quadriceps tendon at closure described by Keblish et al. [4]. This coronally elongated quadriceps tendon was beneficial for better patellar tracking. Next, ITB was released at Gerdy’s tubercle in the early stage if the tibia was externally rotated. This release eliminates the difficulty shifting or everting the patella medially. We also inserted two Kirschner wires of 2 mm in diameter into the tibial tubercle to prevent avulsion fracture at patellar eversion.

**Table 1:** Comparisons of preoperative variables in medial approach group and lateral approach group

	Lateral approach	Medial approach	
Age (years)	63 ± 6.6	66 ± 6.7	ns
Height (cm)	154 ± 5.1	154 ± 6.4	ns
Weight (kg)	54.5 ± 8.9	53 ± 9.8	ns
Preoperative mechanical axis (°)	13.3 ± 5.9	14 ± 6.5	ns
Preoperative flexion contracture (°)	12.7 ± 11.5	13 ± 11.3	ns
Preoperative flexion angle (°)	112.5 ± 14.8	113 ± 17.1	ns
Preoperative KSS (points)	35.3 ± 12.6	36 ± 11.1	ns

All data were exhibited as mean ± SD (range)

*ns* no significant difference

A Scorpio NRG^®^ posterior-stabilized prosthesis (Stryker Howmedica Osteonics, Allendale, NJ, USA) was used for all knees. All surgeries were conducted by a senior surgeon (HS). After bone cuts were completed with an independent cut method, a tensor was used in extension and 90° of flexion to apply a 40-lb distraction force, and release of lateral-side soft tissues was conducted in stages until satisfactory balance was acquired. The patella was replaced in all cases, and all components were fixed with bone cement. Postoperative evaluation was performed at a mean of 43 ± 12 months (18–63 months) after surgery. We compared the following measures between medial approach group and lateral approach group; complications among perioperative time; and surgical time, number of soft-tissue release procedures for balancing during the surgeries, alignment on standing radiography, ROM, KSS at preoperatively and postoperatively, and varus/valgus laxity at immediately after the surgery, and at final follow-up. Measurements of ROM and KSS were taken by one of the authors who did not know which surgical approach had been applied for the patients.

Postoperative coronal laxity was assessed by stress radiographs of the knees using Telos SE arthrometer^®^ (Fa Telos; Medizinisch–Technische, Greisheim, Germany) following a previously reported method [13]. Anteroposterior radiographs were taken while the device was used. Valgus or varus forces of 7 kg to the knees were applied just above the joint on the lateral or medial femoral condyle, whereas the proximal thigh and middle leg were held by the counter supports at 15 flexion. On the stress radiographs, the angle between a line in contact with the bottom of the femoral prosthesis and a line in contact with the upper surface of the tibial prosthesis was measured. We defined this as the valgus angle or varus angle according to the description by Yagishita et al. [12]. These angles indicated the values of medial ligamentous laxity or lateral ligamentous laxity, respectively. The study was approved by the Ethics Committee of Jichi Medical University Hospital, and all patients provided written informed consent.

Statistical assessment included unpaired *t* test to clarify the difference of variables between medial approach group and lateral approach group. Differences in *p* values less than 0.05 were considered statistically significant. A sample size power analysis was performed based on our pilot study and showed that 18 patients in each group would be required to show a difference in a mean of 10 degrees in postoperative flexion with the effect size = 1, test of significance level = 0.05, SD = 10, and a power of test = 80 %.

## Results

All results are summarized in Table 2. There was one case of skin necrosis in the group of lateral approach and one case of superficial infection in the group of medial approach for complication. Both cases were treated successfully. No serious complication such as deep infection or fracture was encountered in either group. No difference was found in all values except for number of soft-tissue release procedures for balancing during the surgeries, and postoperative ROM.

**Table 2:** Comparisons of postoperative variables in medial approach group and lateral approach

	Lateral approach	Medial approach	
Follow-up periods (months)	43.3 ± 14.2	43.2 ± 8.4	ns
Complications	Skin necrosis 1	Superficial infection 1	
Surgical time (min)	133 ± 24	131 ± 18	ns
Number of soft-tissue release procedures	1.5 ± .09	2 ± 0.9	ns
Postoperative mechanical axis (°)	1.6 ± 1	1 ± 1.8	ns
Postoperative flexion contracture (°)	2.9 ± 4.1	2 ± 5.2	ns
Postoperative flexion angle (°)	123.8 ± 11	109 ± 14.3	*p* < 0.001
Postoperative KSS (points)	89.3 ± 4.2	87 ± 4	ns
Varus laxity immediately after surgery (°)	5.5 ± 3.1	5 ± 3	ns
Varus laxity immediately after surgery (°)	4.8 ± 1.8	4.4 ± 1.7	ns
Varus laxity at follow-up (°)	3.7 ± 1.7	4 ± 2	ns
Valgus laxity at follow-up (°)	4 ± 1.9	4 ± 1.9	ns

All data were exhibited as mean ± SD (range)

*KSS* Knee society score; *ns* no significant difference

Averagely, two release procedures were necessary in the groups of medial approach and 1.5 procedures in the group of lateral approach (*p* = 0.14). In the medial approach group, 100 % of cases required ITB release at Gerdy’s tubercle, 83 % required ITB release at the joint level, 21 % required LCL release, and 17 % required POP release. In the lateral approach group, 88 % required ITB release at Gerdy’s tubercle, 46 % required ITB release at the joint level, 13 % required LCL release, and 4 % required PT release.

Postoperative ROM at the follow-up was superior in the lateral approach group. Although there was no difference at flexion contracture angle in two groups, marked difference was found at flexion angle 123.8° in the group of lateral approach versus 109° in the group of medial approach (*p* < 0.001).

## Discussion

Although the rationality of the lateral approach for the valgus deformed knee has been recognized, differences in clinical results between medial and lateral approaches have been uncertain. Hay et al. [10] had randomly divided 32 patients into two groups, in one of which the lateral subvastus approach combined with a tibial tubercle osteotomy and in the other the medial parapatellar approach. And they compared the ROM, a visual analog satisfaction score, the Western Ontario McMasters University Osteoarthritis index, and the KSS at 2 years after the surgery. No significant differences were found between the groups in any of the parameters for clinical outcome. They found significant better patellar tracking in the group of lateral subvastus approach combined with a tibial tubercle osteotomy. Due to complications related with tibial tubercle osteotomy and longer surgical time (10–15 min) in the lateral approach with osteotomy, they did not support its routine use of the except for the patients in whom problems with patellar tracking were anticipated. Hirschman et al. [11] also compared the two groups, the group of lateral parapatellar approach with tibial tubercle osteotomy and medial parapatellar approach group in clinical outcomes. They found better flexion of the knee, better VAS, higher patient’s satisfaction, and longer pain free walking distance in the group of lateral parapatellar approach with tibial tubercle osteotomy at 2 years follow-up. But the revision rate in the group of lateral parapatellar approach with tibial tubercle osteotomy (4 %) was higher than in the group of medial approach (1.5 %), which was mainly due to two cases of traumatic secondary displacement of the tibial tubercle and need for refixation.

Our findings indicate that postoperative knee flexion after TKA in valgus knee was better with a lateral approach than with a medial approach as shown by Hirschman et al. [11]. Thus, lateral approach had some advantages in postoperative clinical outcomes; however, tibial tubercle osteotomy could be problematic procedure.

In the valgus knee, lateral structures are shortened [14]. Due to contracture of the lateral patellar retinaculum, lateral subluxation of the patella is a common problem [14]. Conversely, medial structures are lax or stretched in many instances. Stern et al. [15] reported the difficulty to acquire good coronal balance of TKA by medial approach in 134 valgus deformed knees. He reported only 71 % of valgus deformed knee rated as excellent compared with the 88 % excellent results achieved in an earlier study from the same institution [15]. A lateral approach can directly adjust the tight structure, guarantee good patellar tracking, and reduce vascular compromise to the patella compared with a medial approach with lateral patellar release [16]. Keblish et al. [16] reported good/excellent result in 79 valgus deformed knee treated with lateral approach. And he recommended the lateral approach as the “approach of choice” for fixed valgus deformity in TKA.

However, problems with the lateral approach include difficulty in eversion of the patella [3], and less familiarity for many surgeons [3]. The patellar tendon must not be avulsed, since this is potentially disastrous [17]. To avoid this eventuality, Keblish et al. [16] and Buechel [2] recommended deliberate osteotomy of the tibial tubercle. However, some complications had been reported at the osteotomy of the tibial tubercle [10, 11], and postoperative rehabilitation might have to be delayed to some extent after osteotomy. Fiddian et al. [3] could evert the patella without the osteotomy of the tibial tubercle in lateral approach. Following their methods, we successfully performed TKA using a lateral approach without osteotomy in all cases. To facilitate the eversion of the patella in lateral approach, Boyer et al. [18] completely released the iliotibial band from the Gerdy’s tubercle. In fixed valgus deformity, contracture of ITB is one of the most important factors of excessive external rotation of the tibia. In the condition with the external rotation of the tibia, to evert the patella medially is extremely difficult and dangerous for avulsion of tibial tubercle or rupture of patellar tendon at forceful eversion. Release of ITB in early stage was a crucial key for success in lateral approach. The other details of the surgical tips those we used were mentioned in the part of surgical procedure.

Due to the direct approach for diseased lateral soft tissue, numbers of the release procedure might logically be reduced with a lateral approach compared with a medial approach. Although we could not find the significant difference, our data regarding the relatively less number of release procedures in the group of lateral approach compared with the group of medial approach indicated the rationality of lateral approach to adjust the soft-tissue balance in valgus deformed knee. We could confirm by the stress radiograph that the similar coronal balance could be achieved with reduced number of release procedures in lateral approach group.

To explain the reason underlying the better ROM with a lateral approach than with a medial approach, we paid attention to differences in attachments of the vastus lateralis and vastus medialis. The vastus medialis attaches at the midpoint of the patella or more distally, whereas the vastus lateralis attaches at the proximal patella [19]. Even with the same length of parapatellar dissection, invasiveness to the muscle would differ between medial and lateral approaches. Reduced invasiveness to the quadriceps muscle and reduced release procedures for lateral structures with a lateral approach could result into better postoperative ROM.

Niki et al. [20] compared the clinical outcomes and perioperative data, including total blood loss, operative time, myoglobin/creatinine phosphokinase (CPK) index, and VAS score for pain between 26 patients with valgus knee who underwent MIS–TKA using a lateral subvastus approach and 26 patients treated with MIS–TKA by medial approach. Although they did not mention about the detail of postoperative ROM, they found the comparable clinical scores, radiographic accuracy, and postoperative complication rate. From reduced serum myoglobin index on postoperative day 1 in the group of lateral subvastus approach, they showed the muscle-sparing effect in the lateral approach. And from lower VAS score at 7 days after surgery in the group, they made explanation as follows: The subcutaneous nerve plexus on the lateral side of the skin is less developed than on the anterior or medial sides. These subjective and objective findings shown by Niki et al. [20] could be a key to explain the reason for better flexion angle found in lateral approach group.

There were some limitations that need to be addressed regarding the present study. The first limitation concerned about the relatively small number of the patients in both groups. To achieve the firm conclusion, we will continue the study. The second limitation was the shortage of the subjective and objective finding to explain the reason for better flexion angle found in lateral approach group. Measurement of the myoglobin/CPKindex, and VAS score for pain shown by Niki et al. [20], and inflammatory markers (IL-6, IL-10, IL-8 and tumor necrosis factor—*α*) would be beneficial for the study in the future. The third limitation was the duration of the follow-up. In accordance with the improvement of the implants and surgical technique, good clinical result had become common in TKA. Long-term observation over 10 years is necessary to state the real value of the technique. From such point of view, the follow-up period of 43 ± 12 months in present study was short. However, ROM and laxity of the knee joint over 1 year after TKA were relatively constant in many studies, 43 months would be sufficient to compare the ROM and laxity.

In conclusion, TKA using a lateral approach without tibial osteotomy could provide better postoperative ROM compared with knees treated using a medial approach.
